# Hand-assisted laparoscopic surgery using Gelport

**DOI:** 10.4103/0972-9941.18994

**Published:** 2005-09

**Authors:** Puneet Gupta, V K Bhartia

**Affiliations:** Institute of Minimally Invasive Surgery, AMRI Hospitals, Kolkata, India

**Keywords:** Gelport, hand-assisted laparoscopic surgery, hand-assisted laparoscopic surgery colectomy

## Abstract

**Introduction:**

Minimally invasive surgery has revolutionized general surgery during the past 10 years. However, for more advanced surgical procedures, the acceptance of the minimally invasive approach has been slower than expected. Advanced laparoscopic surgery is complex and time-consuming. The major drawbacks of laparoscopic surgery are two-dimensional view, lack of depth perception and loss of tactile sensation. This has led to the innovation of hand-assisted laparoscopic surgery (HALS). The objective of the present study was to determine that safety of HALS.

**Materials and Methods:**

We preformed 18 HALS procedures in our department between July 2003 and January 2005 on patients who had given their informed consent for the use of Gelport. Out of these, 15 were colectomy, 2 nephrectomy and 1 splenectomy. Out of the 18 patients, 13 were males and 5 were females with the age group ranging from 44 to 72 years.

**Results:**

Hand-assisted laparoscopic surgery could be completed in 17 patients maintaining all the oncological principals of surgery. The mean operating times were 120 min for right haemicolectomy, 135 min for left colectomy, 150 min for splenectomy, and 150 min for nephrectomy. The patient undergoing radical nephrectomy by HALS had to be converted to open surgery. As the tumour was large and adherent to the spleen and posterior peritoneal wall. Postoperative recovery was excellent with an average hospital stay of 5 days. Histopathology report showed wide clearance and till date we have a good follow up of 30–380 days.

**Conclusion:**

Hand-assisted laparoscopic surgery allows tactile sensation and depth perception thereby may simplify the complex procedures. This may result in reduction of operating time and conversion rates at the same time maintaining all the oncological principles. Hand-assisted laparoscopic surgery strikes a perfect balance between an extended open laparotomy incision and an excessively tedious laparoscopic exercise. Hand assistance is an initial tool for the trainee laparoscopic surgeon or a last resort for the experienced laparoscopic surgeon.

Minimally invasive surgery has revolutionized general surgery during the past 10 years. However, for more advanced surgical procedures, the acceptance of the minimally invasive approach has been slower than expected.[1] Advanced laparoscopic surgery is complex and time-consuming. The major drawbacks of laparoscopic surgery are two-dimensional (2D) view, lack of depth perception and loss of tactile sensation.

The previous observations led to the development of a device that allows insertion of a hand into the abdominal cavity while maintaining pneumoperitoneum, enabling the surgeon to perform the operation according to the principles of open surgery while retaining many of the advantages of a laparoscopic procedure. Although it is an aggressive procedure, HALS preserves the features of the minimally invasive approach, maintains all the oncological principles.[2]

The objective of the present study is to determine that HALS is safe and is a good connecting link between total laparoscopic procedures and conventional open technique.

## MATERIALS AND METHODS

We preformed 18 HALS procedures in our department between July 2003 and January 2005 on patients who had given their informed consent. All the operations were performed by the same surgical team. Out of the 18 patients, 13 were males and 5 were females with the age group ranging from 44 to 72 years.

In all patients a thorough history was obtained and clinical examination performed. Investigations included routine blood tests, liver function tests, renal function tests and abdominal sonography. In patients who were due to undergo a colectomy a lower GI endoscopy was done to exclude any other lesion. Patients undergoing nephrectomy underwent an intravenous urogram to assess the function of the other kidney and a CT scan was done for any evidence of mass lesion or extension of thrombus into the IVC. All patients received bowel preparation prior to surgery. The patient undergoing splenectomy received vaccination. Routine thromboembolic prophylaxis was administered in all patients.

### Technique of HALS

Hand-assisted laparoscopic surgery involved the insertion of a surgeon's hand inside the abdominal cavity via a small incision. Average incision size to introduce a hand was 7.0 cm corresponding to the surgeon's glove size. The exact positioning of the handport depends on various factors such as surgeon's dexterity, comfort and target area.

We have used the Gelport as a device of handport because of its ease of use, reliable maintenance of pneumoperitoneum and ability to rapidly switch over to conversion if necessary.

Gelport[3,4] is a sterile, disposable food and drug administration – approved hand access device intended for single use. The device consists of

- A wound-protecting sheath;
- A wound retractor;
- A unique gel seal cap.

The wound-protecting sheath is designed to protect incisions and soft tissues from malignant and infectious exposure. The wound retractor facilitates extracorporeal resection and organ removal while minimizing the incision size. The gel seal cap enables unlimited exchange of hand and instruments while maintaining pneumoperitoneum.

After creating the pneumoperitoneum, laparoscopic assessment of the whole abdomen was done via a telescope introduced through the umbilical port. It is important to plan the placement of the hand port based on the organ being targeted at surgery. It is better to place the handport some distance away from the area of pathology following the principle of triangulation of the ports, so that there is enough working space. Also, the possibility of conversion to an open procedure and the need for extension of this mini laparotomy incision to a full laparotomy incision should be kept in mind.

### Right colectomy

After creating pneumoperitoneum, the peritoneal cavity was inspected to exlclude liver or peritoneal metastasis or ascites. A 7-cm infraumbilical midline incision was made for the placement of Gelport. Gelport was fixed as discussed above. The operating surgeon, standing on the left side of the patient, inserted his nondominant (left) hand in to the abdomen and palpated the lesion. The ascending colon was mobilized gradually from the caecum towards the hepatic flexure – the counter traction being applied by the surgeon's hand. Gradually, the colon was mobilized towards the midline preserving the ureter, gonadal vessels and duodenum. A 5-mm port was made in the epigastrium for the right hand of the surgeon that operated a Harmonic shears (Ethicon Endosurgery, Cincinati, USA). After adequate mobilization, the right colon containing the pathology was exteriorized through the handport site and resected. An end-to-end, single layer, hand-sewn ileo-transverse anastomosis was performed. The anastomosed gut was placed back in the peritoneal cavity. The handport site was closed with 10 vicryl after ensuring haemostasis.

### Left colectomy

After preliminary laparoscopy, a midline supraumbilical port was made for Gelport. The surgeon inserted his left hand and mobilization of the left colon started from the splenic flexure downwards. After completing the mobilization of the descending and sigmoid colon, the area of pathology was exteriorized. Segmental resection with end-to-end anastomosis was performed extracorporealy and the anastomosed bowel was reposited into the abdominal cavity.

### Left-sided nephrectomy

After doing a preliminary laparoscopy through a 5-mm umbilical port, a 7-cm supraumbilical midline incision was made for the Gelport. A 10-mm working port for introduction of the operating instrument was made in the midclavicular line two fingers above the level of the umbilicus. The surgeon standing on the right side of the patient inserted his nondominant hand and retracted the splenic flexure of the colon, and the kidney was mobilized along with Gerota's fascia. The left colon was further mobilized; the ureter was identified, looped, clipped and divided. The proximal ureter was traced upwards towards the hilum. The superior and lateral attachments of the kidney were removed with the Harmonic shears. Renal vessels were dissected separately and with the help of the hand the renal artery and vein were ligated individually and divided. The specimen was released medially and posteriorly and put in an endobag. The renal fossa was inspected for haemostasis, and the specimen retrieved through the handport incision site. The incision site was closed after putting a drain in the renal fossa.

### Splenectomy

After creating the pneumoperitoneum, a midline supraumbilical 7-cm incision was made for the Gelport about two fingers above the umbilical port. The surgeon inserted his left hand through the hand port to help in mobilization of the spleen starting from its inferior pole. The right hand working port was placed just outside the left midclavicular line about 2 cm above the umbilicus (as in standard laparoscopic splenectomy). The splenic vessels were approached through the lesser sac and were dissected, ligated individually and divided. The spleen was freed from other ligamentous attachments. It was then placed into a home-made extraction bag with the help of the hand and extracted through the handport.

## RESULTS

We selected 18 patients for HALS and were able to complete the HALS procedure in 17 patients. One procedure was converted to the conventional open method. Indications for the HALS procedure are shown in [Table 1].

**Table 1:** Indications of HALS

Procedure	Indication	No. of procedures
Colectomy	Benign	5
	Malignant	10
Nephrectomy	Hydronephrosis	1
	Carcinoma	1
Splenectomy	Splenic abscess	1
Total		18

Among the colectomy group, benign indications included three cases of ileocaecal tuberculosis, one case of diverticultis and one case of bleeding ascending colon polyp. The malignant group included neoplasia involving the right colon in seven patients and the left colon in three patients.

The various types of procedures, which we performed, are shown in [Table 2].

**Table 2:** Hand-assisted laparoscopic surgery procedures performed

Right hemicolectomy	11
Left hemicolectomy	4
Nephrectomy left	1
Splenectomy	1
Total	17

Hand-assisted laparoscopic surgery colectomy could be completed in reasonably good time maintaining all the oncological principals of surgery. Hand-assisted laparoscopic surgery nephrectomy in a case of renal cell carcinoma had to be converted due to large adherent tumour mass, which was adhered to the spleen and posterior peritoneal wall.

The complications, which we experienced, are shown in [Table 3].

**Table 3:** Complications

Conversion	1
Wound-related problems	3
Air leak	1
Hand fatigue	4

The mean operating time was 120 min for the right hemicolectomy (range: 90–210 min), 135 min for left hemicolectomy (range: 100–220 min), 150 min for nephrectomy and 150 min for splenectomy. The mean operating times for the procedures were comparable to the those required for the open counterparts; however, we have not compared it directly in our series.

Postoperative recovery was excellent with an average hospital stay of 5 days. Blood transfusion was not required in any patient undergoing HALS except in the case of nephrectomy where we had to convert to the conventional open technique. In the colectomy group, the patients were commenced on oral feeds from postoperative day 3 and patients were discharged in 5–7 days. The nephrectomy patient was commenced on oral feeds on the first postoperative day and was discharged on the fifth day. The splenectomy patient was discharged on fourth postoperative day.

In procedures for malignancy, histopathology report showed wide clearance and till date we have a good follow up of 30–380 days.

## DISCUSSION

In spite of the advancement in instrumentation and standardization of procedures, advanced laparoscopic procedures are still limited to the major centres and specialist laparoscopic surgeons. There are a number of reasons for this, including lack of adequate training, lack of instrumentation, lack of tactile sensation and depth perception and fear of changing the tissue biology in malignancies.[5,6]

The various drawbacks of total laparoscopic approach can be summarized as:

- Lack of normal stereoscopic vision (2D view).
- Reduced tactile feedback and depth perception.
- Inadequate exposure/locoregional assessment, specially in cancer surgery.
- Excessive *instrument traffic* causing increased rate of infection/port site metastasis.
- Multiple access ports thus increasing the risk of iatrogenic injury.
- Limitation in specimen retrieval, specially cancer tissue and risk of port site metastasis.
- Difficulty in executing complex anastomosis and the cost involved in the stapling devices.

As advanced procedures have developed, some authors have proposed the concept of HALS [7]–[9] a technique that was, for the most part, rejected by the surgical community because it violated the fundamental principle of minimal invasion.

Direct manual manipulation through the tight fascial incision during laparoscopy was reported in early 1990[10] and pneumoperitoneum was maintained by apposing the wound edges and applying surgical sponges around the incision.[11] But these methods hampered the mobility of the surgeon's hand and were unable to prevent air leak. This led to the innovation of various types of handports like lap disc, pneumosleeve and Gelport, etc.[12] The sine quonon of any laparoscopic assisted device is that it retains the pneumoperitoneum.

The rationale of HALS is that an incision for retrieval of the specimen can be made at the commencement of the surgery instead of making it at the end so that it can be used favourably for placing a hand for providing intraoperative assistance. This increases the surgeon's efficiency at the same time preserves the advantages of minimal access surgery.[13] The main role is to help in difficult cases before conversion is necessary or for training unskilled surgeons, and not as an alternative to pure laparoscopic surgery.[14]

The obvious advantage of HALS is that the surgeon regains direct tactile feedback and acquires improved hand eye coordination thereby increasing ‘surgical action efficiency’. The recovery of tactile feeling shortens certain dissection manoeuvres, avoids unnecessary movements, favours the smooth traction and exposures of structures, and facilitates the control of unexpected or difficult situations.[15] The tactile feedback afforded by the surgeon's hand within the abdomen psychologically inspires confidence in the neophyte laparoscopist.[16] The hand-assisted technique appeared to be useful in minimally invasive colorectal surgery, splenectomy for splenomegaly, living-related donor nephrectomy, and procedures considered too complex for a laparoscopic approach.[17,18] Perhaps the most suitable operations for HALS are those that require extraction of a specimen and therefore necessitate an incision anyways.

As far as malignant disease is concerned, the advantages that HALS offers are the detection of metastatic lesions and the local stage of tumors.[19] It simplifies the performances of difficult procedures for experienced surgeons and can initiate less experienced surgeons in advanced laparoscopic surgery. Port-site recurrence has been a major argument against laparoscopic surgery for malignant lesions. Results of recent trials, however, demonstrate that wound recurrence in open surgery and port site recurrence in laparoscopic surgery have an equal probability.[20,21] Hand-assisted laparoscopic surgery further reduces the risk as there is a wound-protecting sheath in the handport devices and the tissues do not directly come in contact with the wound.[22,23]

The main drawback of HALS is that it requires an additional incision, thus increasing trauma. Furthermore, the hand takes up space inside the abdomen and may hamper certain manoeuvres, and may also induce hand fatigue in prolonged procedures.[24] The choice of the incision site is likely to depend on whether the intra-abdominal hand is the surgeon's or the assistant's. If the surgeon uses his/her nondominant hand the assistant's, dominant hand is used. If the surgeon's hand is introduced, it should not impair the vision provided by the telescope.[25] The best way to prevent hand fatigue is careful choreography and planning of trocar and handport placement. The location of incision depends on the target organ and on the surgeon's dexterity. The basic principle of instrument triangulation is valid for HALS with the hand being considered as an instrument.[26] For precise planning of the procedure, laparoscopy should always precede placement of the handport incision. The handport incision should never be placed directly over the target area. Finally, the handport incision should be planned so that turning it into full conversion incision can be done with a simple extension, if necessary.

In our series, though a small one, we had to convert to open surgery in one patient, probably as a result of improper case selection. We had minor wound infection in three patients, which subsided with conservative management. Maartense et al. have reported that they did not have any wound infection in their series of 150 patients undergoing HALS.[23] We reused the Gelport device after proper resterilization with ethylene oxide. The practice of reuse of single-use medical devices has been prevalent in many other areas. For example ERCP accessories, angiography guidewires, and even disposable laparoscopic instruments have been reused after proper sterilization without detrimental effect in terms of increased the risk of infection.[27–30]

As our series is small we have not compared our data with the larger series from the literature but our mean operating times were comparable to the various studies (130 and 150 min for colectomy, splenectomy and nephrectomy, respectively in our series and 158, 100 and 155 min as reported by Maartense et al,[23] 140, 90 and 175 min, respectively reported by Chowbey et al.[13]

Hand-assisted laparoscopic surgery may allow surgeons to perform more complex operations by offering the surgeon the advantages of tactile feedback, safe retraction and the ability to perform blunt dissection. We believe that this will become a part of the surgeon's armamentarium and will be incorporated into the modern paradigm of laparoscopic surgery.

## CONCLUSION

Hand-assisted laparoscopic surgery converts a 2D operation into a 3D one by adding tactile sensation and depth perception thereby simplifying the complex procedures, it reduces the operating time, reduces conversion rate, without increasing the cost, and at the same time maintains all the oncological principles. Hand-assisted laparoscopic surgery strikes a perfect balance between an extended open laparotomy incision and an excessively tedious laparoscopic exercise. Hand assistance is an initial tool for a trainee laparoscopic surgeon or the last resort for an experienced laparoscopic surgeon.
